# Comparison of clinical outcomes of single-incision versus multi-port laparoscopic surgery for descending colon cancer: a propensity score-matched analysis

**DOI:** 10.1186/s12876-022-02597-z

**Published:** 2022-12-09

**Authors:** Mitsuyoshi Tei, Yozo Suzuki, Toshinori Sueda, Kazuya Iwamoto, Atsushi Naito, Masatoshi Nomura, Yukihiro Yoshikawa, Masahisa Ohtsuka, Mitsunobu Imasato, Tsunekazu Mizushima, Hiroki Akamatsu

**Affiliations:** 1 Department of Surgery, Osaka Rosai Hospital, 1179-3 Nagasone-cho, Kita-ku, Sakai, 591-8025 Japan; 2 Department of Surgery, Toyonaka Municipal Hospital, Toyonaka, Japan; 3 Department of Surgery, Osaka Police Hospital, Osaka, Japan; 4 Department of Surgery, Osaka Minato Central Hospital, Osaka, Japan

**Keywords:** Single-incision laparoscopic surgery, Descending cancer, Outcome

## Abstract

**Background:**

The clinical impact of single-incision laparoscopic surgery (SILS) for descending colon cancer (DCC) is unclear. The aim of this study was to evaluate the clinical outcomes of SILS for DCC compared with multi-port laparoscopic surgery (MPLS).

**Methods:**

We retrospectively analyzed 137 consecutive patients with stage I–III DCC who underwent SILS or MPLS at two high-volume multidisciplinary tertiary hospitals between April 2008 and December 2018, using propensity score-matched analysis.

**Results:**

After propensity score-matching, we enrolled 88 patients (n = 44 in each group). SILS was successful in 97.7% of the matched cohort. Compared with the MPLS group, the SILS group showed significantly less blood loss and a greater number of harvested lymph nodes. Morbidity rates were similar between groups. Recurrence pattern did not differ between groups. No significant differences were found between groups in terms of 3-year disease-free and overall survivals.

**Conclusion:**

SILS appears safe and feasible and can provide satisfactory oncological outcomes for patients with DCC.

## Introduction

Single-incision laparoscopic surgery (SILS) represents a recent advance in minimally invasive techniques. The first case of SILS was described for right colectomy in 2008 [1]. The benefits reportedly included better cosmetic outcomes, less postoperative pain, faster postoperative recovery, and earlier discharge from the hospital compared to multi-port laparoscopic surgery (MPLS) [2–5]. In several retrospective studies, SILS has been identified as a feasible and safe method of treating colon cancer in terms of both short- and long-term oncological outcomes [5–7]. In recent randomized controlled trials comparing SILS with MPLS, SILS has been shown to be equivalent to MPLS in term of short-term outcomes and can be considered an option for selected patients with colon cancer [8–10].

However, cases of descending colon cancers (DCC) were excluded from the above retrospective [5, 6] and randomized studies [8–10] because of technical difficulties, particularly mobilization of the splenic flexure, and judgment of the area for lymph node dissection due to the anatomical complexity of the region. The impact of SILS on DCC is unclear. The aim of this study was thus to evaluate the clinical outcomes of SILS for DCC compared with MPLS in our institutions.

## Patients and methods

### Patient populations and surgeons

Consecutive patients who underwent laparoscopic surgery (including MPLS and SILS) for DCC between April 2008 and December 2018 at Osaka Police Hospital and Osaka Rosai Hospital were assessed. Cases of obstruction or perforation that required emergent operation were excluded from this study.

The first case of SILS for DCC was carried out in March 2011. Since then, the indications for SILS have gradually been expanded to include advanced cancers. Patients received a sheet describing the differences between MPLS and SILS, and also received a thorough explanation of each operative procedure. All patients agreed to undergo SILS, and provided written informed consent.

### Lymphadenectomy for DCC according to the tumor location

According to the Japanese Society for Cancer of the Colon and Rectum Guideline for the Treatment of Colorectal Cancer [11], D2 lymph node dissection was performed for clinical T1 tumor and D3 lymph node dissection for clinical T2 or greater tumors. In principle, at least 10 cm of normal bowel both proximal and distal to the tumor was resected. For patients with tumor located in the proximal one-third of the descending colon [12, 13], we performed left hemicolectomy with D3 lymphadenectomy, which involves complete dissection of the pericolic lymph nodes (node station 221, 231, and 241), intermediate lymph nodes (nodes 222, 232, 242, and 252), main lymph nodes (node 223) along the middle colic artery (MCA), and main lymph nodes (node 253) along the inferior mesenteric artery (IMA) as defined by the Japanese Society for Cancer of the Colon and Rectum [14]. On the other hand, segmental colectomy was performed for DCC located in the distal two-thirds of the descending colon [12, 13]. In segmental colectomy, D3 lymphadenectomy involves complete dissection of regional lymph nodes, including the pericolic lymph nodes (nodes 221, 231, and 241), intermediate lymph nodes (nodes 232, 242, and 252), and main lymph nodes (node 253) along the IMA as defined by the Japanese Society for Cancer of the Colon and Rectum [14].

### Surgical technique

In this study, SILS was performed by three surgeons, while MPLS was performed by five surgeons. We used four or five ports for MPLS, including a camera port. In contrast, for SILS, a single, 30-mm intra-umbilical incision was made and an E-Z Access port device (Hakko, Nagano, Japan) was placed on the Lap Protector™ (Hakko) for insertion of two 5-mm trocars and one 12-mm trocar into an equilateral triangle, as described previously [7, 15]. With the patient in a Trendelenburg position with the left side elevated, the sigmoid mesocolon was mobilized from the retroperitoneal plane using a medial-to-lateral approach. After identifying the left ureter and gonadal vessels, the IMA and left colic artery were exposed. The LCA and inferior mesenteric vein (IMV) were divided at the root after radical lymphadenectomy along the IMA, preserving the superior rectal artery. The descending mesocolon was mobilized from the retroperitoneal planes, including Gerota’s fascia, using a medial-to-lateral approach, up to the dorsal surface of the pancreas. Next, changing the patient to a reverse Trendelenburg position with left side elevated, the greater omentum was separated from the transverse colon, the omental bursa was opened, and the inferior border of the pancreas was exposed. The transverse mesocolon was separated from the inferior border of the pancreas. Following these procedures, the splenocolic ligament and lateral attachment of the descending colon were divided, and the splenic flexure was fully mobilized. The IMV was again divided at the inferior border of the pancreas. In left hemicolectomy, the left branch of the MCA was also divided. Finally, the transverse and sigmoid colon, including the DCC, was pulled out through a small incision at the umbilicus and transected using linear staplers. A functional end-to-end anastomosis was then created extracorporeally. No drains were used. The single skin incision was closed using absorbable sutures.

### Data collection

Patient age, sex, body mass index (BMI), Eastern Cooperative Oncology Group performance status (ECOG-PS), American Society of Anesthesiologists (ASA) score, previous abdominal surgery, clinical TNM classification, and comorbidities were obtained from the medical records. As listed in Table 1, cardiac disease consisted of ischemic disease, chronic heart failure or cardiomyopathy. Pulmonary disease consisted of asthma, chronic obstructive pulmonary disease or interstitial pneumonia. Cerebrovascular disease consisted of a history of transient ischemic attacks or cerebrovascular events, with or without neurological deficit. Postoperative complications were classified according to the Clavien-Dindo classification [16]. Infectious complications consisted of abscess, colitis, urinary tract infection, nephritis, catheter-related infection, or cholecystitis. Operative mortality was defined as death during the same admission or within 30 days of surgery. All patients were followed-up for at least 30 days after surgery. This study was approved by the institutional review boards at Osaka Police Hospital (approval no. 1468) and Osaka Rosai Hospital (approval no. 2021-82).

**Table 1:** Demographic characteristics of patients

	Overall (N=137)			Propensity score-matched pairs (n=88)		
	Lap (n=85)	SILS (n=52)	*P* value	Lap (n=44)	SILS (n=44)	*P* value
Age, years, median (IQR)	71 (63-78)	69 (63-74)	0.444	67 (57-75)	69 (60-74)	0.551
Sex, male, n (%)	57 (67.1)	27 (51.9)	0.104	19 (43.2)	19 (43.2)	1
BMI, kg/m^2^, median (IQR)	23.0 (20.9-24.9)	23.1 (20.9-25.1)	0.982	22.8 (20.0-25.5)	23.3 (20.9-25.0)	0.679
ECOG-PS, 0 or 1, n (%)	84 (98.8)	50 (96.2)	0.557	43 (97.7)	43 (97.7)	1
ASA score, 1 or 2, n (%)	72 (84.7)	42 (80.8)	0.639	37 (84.1)	36 (81.8)	1
Previous abdominal surgery, n (%)	17 (20)	17 (32.7)	0.107	12 (27.3)	11 (25)	1
Clinical TNM stage, n (%)			0.456			0.906
I	33 (38.8)	15 (28.9)		17 (38.6)	15 (34.1)	
II	26 (30.6)	17 (32.7)		14 (31.8)	15 (34.1)	
III	26 (30.6)	20 (38.5)		13 (29.6)	14 (31.8)	
*Comorbidities, n (%)						
Cardiac	11 (12.9)	8 (15.4)	0.800	2 (4.6)	6 (13.6)	0.266
Pulmonary	13 (15.3)	7 (13.5)	0.809	6 (13.6)	6 (13.6)	1
Diabetes	19 (22.4)	7 (13.5)	0.263	8 (18.2)	5 (11.4)	0.549
Cerebrovascular	8 (9.4)	9 (17.3)	0.191	4 (9.1)	7 (15.9)	0.521
Anticoagulant therapy	12 (14.1)	9 (17.3)	0.632	3 (6.8)	8 (18.2)	0.196

*ECOG-PS* Eastern Cooperative Oncology Group Performance Status Scale, *ASA score* American Society of Anesthesiologists Score, *BMI* body mass index, *Comorbidities: Cardiac = ischemic disease, chronic heart failure and cardiomyopathy, excluded hypertension; Pulmonary = asthma, chronic obstructive pulmonary disease, and interstitial pneumonia Cerebrovascular = history of transient ischemic attacks and cerebrovascular event with or without neurological deficit

### Statistical methods

**Fig. 1:**
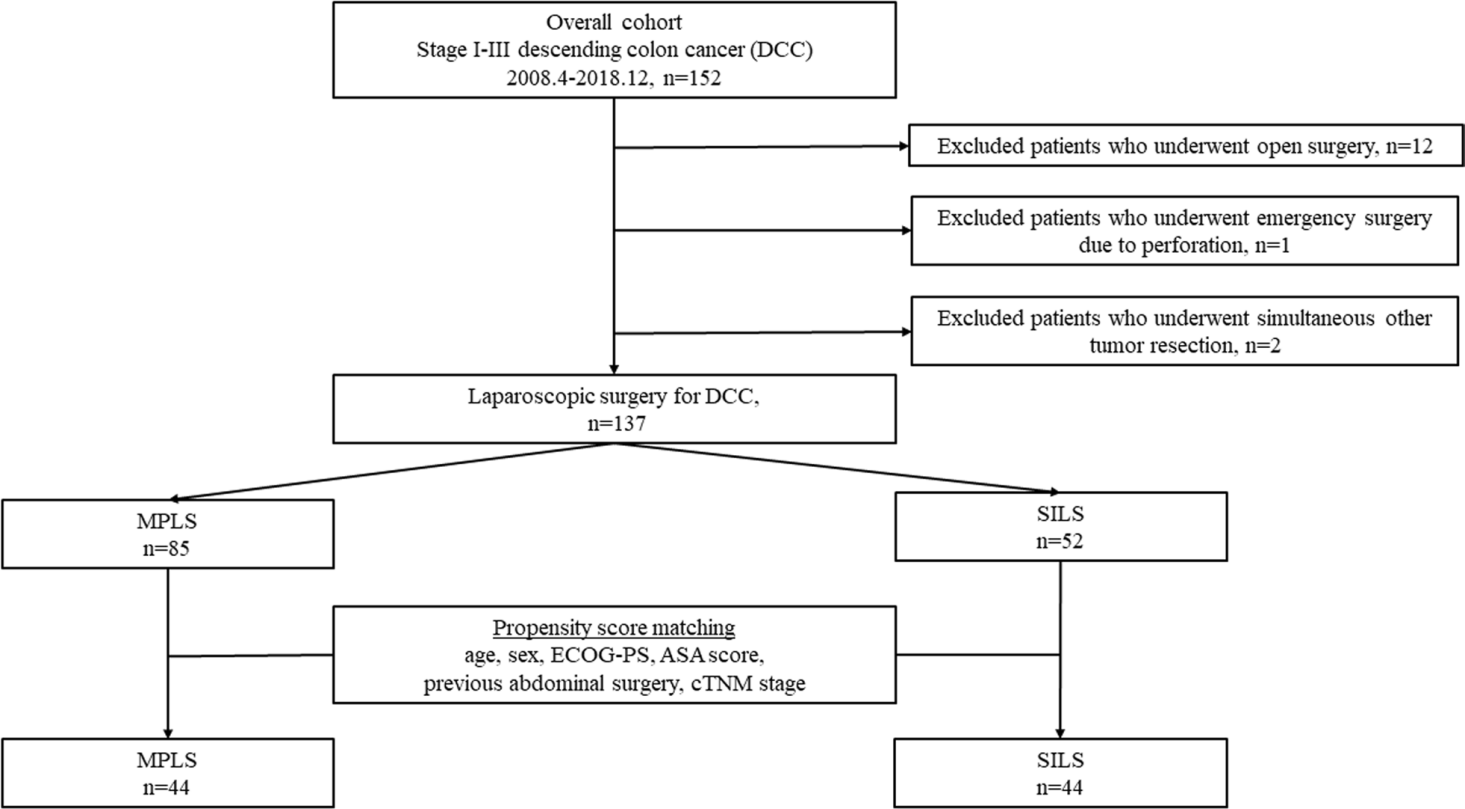
Flowchart of patients who underwent SILS or MPLS for DCC, describing the patient-matching process

Prior to propensity score-matching, the t test or Wilcoxon rank-sum test was used for continuous variables, and the χ^2^ test or Fisher’s exact test was applied for categorical variables. Propensity score-matching was then applied to minimize the possibility of selection bias and to adjust for significant differences in the baseline characteristics of patients (Fig. 1). The first step in the matching process was to complete a multivariate logistic regression analysis to obtain propensity scores. The following nine covariates that might affect short- and long-term outcomes for SILS were included in the model for calculating the propensity score: age, sex, ECOG-PS, ASA score, previous abdominal surgery, and clinical TNM classification. The next step was the 1:1 matching process, using calipers set at 0.2. This propensity score-matching was used to evaluate the effects of SILS on surgical and pathological outcomes. After propensity score-matching, baseline characteristics, including covariates not entered into the propensity score model, were compared between groups using bivariate analyses.

Data are presented as the median and interquartile range (IQR) for continuous variables and as the frequency and percentage for categorical variables. The χ^2^ test was used for comparisons of categorical variables. Student’s t test was used to determine the significance of differences between continuous variables. Survival curves were calculated using the Kaplan–Meier method and were then compared by log-rank testing. Potential prognostic factors associated with oncological outcome were analyzed by uni- and multivariate analyses. Variables showing values of *P* < 0.20 in univariate analyses were analyzed further by stepwise multivariate analysis using Cox proportional hazards modeling to determine the combination of variables that differed significantly between the two groups. Values of *p* < 0.05 were considered statistically significant. All statistical analyses were performed using JMP version 16.0 software (SAS Institute, Cary, NC, USA).

## Results

### Baseline patient profiles

An overview of our study is shown in Fig. 1. Among 152 consecutive patients who underwent primary tumor resection for DCC, 15 patients were excluded. These exclusions were due to open surgery in 12 patients, emergency surgery due to perforation in 1 patient, and simultaneous resection of another tumor in 2 patients (ascending colon cancer in 1, gastrointestinal tumor in 1). The total sample size was thus 137 patients who underwent SILS (n = 52) or MPLS (n = 85) for DCC. Table 1 lists the demographic characteristics of the overall cohort and for propensity score-matched patients. After matching, 44 matched pairs were selected. Baseline characteristics of patients were conserved between the two matched groups.

### Comparison of short-term outcomes between groups

Table 2 summarizes the details of operative findings between groups. Compared with the MPLS group, blood loss was significantly less in the SILS group both before (p < 0.001) and after matching (p = 0.011). In the overall cohort, D3 lymph node dissection rate was significantly larger in the SILS group before matching (p < 0.001), but was not significant after matching (p = 0.085). In the MPLS group, 1 patient was converted to open surgery because of intraoperative bleeding. In the SILS group, 1 patient required an additional port for development of the operative field. No relevant differences were found between groups in terms of procedure, operative time or multivisceral resection rate before or after matching.

**Table 2:** Operative findings

	Overall (N=137)			Propensity score-matched pairs (n=88)		
	Lap (n=85)	SILS (n=52)	*P* value	Lap (n=44)	SILS (n=44)	*P* value
Procedure			0.158			1
Left hemicolectomy	33 (24.1)	15 (28.9)		12 (27.3)	13 (29.6)	
Segmental colectomy	49 (57.7)	37 (71.2)		32 (72.7)	31 (70.5)	
Subtotal colectomy	3 (3.5)	0				
Blood loss, ml, median (IQR)	30 (5-100)	5 (5-67)	<0.001	26 (5-90)	5 (5-67)	0.011
Operative time, minutes, median (IQR)	227 (191-274)	240 (189-258)	0.678	234 (192-294)	240 (192-262)	0.900
Extent of lymph node dissection, D3, n (%)	41 (48.2)	36 (69.2)	0.021	20 (45.5)	29 (65.9)	0.085
Multivisceral resection, n (%)	6 (7.1)	3 (5.8)	1	1 (2.3)	2 (4.6)	1
Conversion to open surgery, n (%)	1 (1.2)	0	–	1 (2.3)	0	–
Required an additional port, n (%)	0	1 (1.9)	–	0	1 (2.3)	–

Table 3 depicts the postoperative complications that occurred in each group. The rate of Clavien-Dindo grade ≥ 2 events did not differ between groups before or after matching. Two patients in the MPLS group and 1 patient in the SILS group underwent reoperation due to anastomotic leakage. Perioperative death was not found in the overall cohort. Median duration of hospitalization was 10 days in both groups after matching.

**Table 3:** Postoperative complications

Clavien-Dindo classification (Grade ≥ 2), n (%)	Overall (N = 137)			Propensity score-matched pairs (n = 88)		
	Lap (n = 85)	SILS (n = 52)	P value	Lap (n = 44)	SILS (n = 44)	*P* value
Anastomotic leakage	3 (3.5)	1 (1.9)	1	1 (2.3)	1 (2.3)	1
Wound infection	6 (7.1)	4 (7.7)	1	5 (11.4)	3 (6.8)	0.713
Bowel obstruction	2 (2.4)	5 (9.6)	0.105	2 (4.5)	3 (6.8)	1
Pneumonia	1 (1.2)	1 (1.9)	1	1 (2.3)	1 (2.3)	1
*Infectious complications	2 (2.4)	7 (13.5)	0.026	2 (4.5)	5 (11.4)	0.260
Reoperation	2 (2.4)	1 (1.9)	1	0	1 (2.3)	–
Perioperative death	0	0	–	0	0	–
Overall complication	13 (15.3)	13 (25)	0.182	7 (15.9)	10 (22.7)	0.435
Postoperative hospital stay, days, median (IQR)	11 (9–15)	9 (7–13)	0.025	10 (8–14)	10 (7–13)	0.432

*Infectious complications = abscess, colitis, urinary tract infection, nephritis, catheter-related infection, cholecystitis

The pathological features and oncological outcomes are summarized in Table 4. The number of harvested lymph nodes was significantly larger in the SILS group than in the MPLS group, both before (p < 0.001) and after matching (p = 0.043). Tumor size, proximal margin, distal margin, tumor invasion, lymph node metastasis, pathological TNM classification, and number of patients who received adjuvant chemotherapy were similar in both groups. Radial margin positivity was not found in any patients.

**Table 4:** Pathological features and oncological outcomes

	Overall (n = 137)			Propensity score-matched pairs (n = 88)		
	Lap (n = 85)	SILS (n = 52)	*P* value	Lap (n = 44)	SILS (n = 44)	*P* value
Tumor size, mm, median (IQR)	25 (16-45)	40 (26-50)	0.038	25 (15-50)	38 (23-45)	0.128
Proximal margin, mm, median (IQR)	105 (95-110)	110 (105-118)	0.749	105 (90-115)	110 (95-110)	0.648
Distal margin, mm, median (IQR)	95 (80-110)	90 (85-108)	0.545	95 (88-110)	90 (85-110)	0.701
Number of harvested lymph nodes, median (IQR)	14 (8-22)	21 (12-29)	<0.001	14 (8-22)	20 (11-27)	0.043
Tumor invasion, n (%)			0.353			0.568
pT1	25 (29.4)	10		14 (31.8)	10	
pT2	12 (14.1)	(19.2)			(22.7)	
pT3	34 (40)			6 (13.6)		
pT4	14 (16.5)	5 (9.6) 28 (53.9) 9 (17.3)		16 (36.4) 8 (18.2)	4 (9.1) 22 (50) 8 (18.2)	
Lymph node metastasis, n (%)			0.251			0.086
pN0	62 (72.9)	31 (59.6)		33 (75)	26 (59.1)	
pN1a	11 (12.9)	7 (13.5)		6 (13.6)	7 (15.9)	
pN1b	6 (7.1)	8 (15.4)		1 (2.3)	6 (13.6)	
pN2a	5 (5.9)	3 (5.8)		4(9.1)	2 (4.6)	
pN2b	1 (1.2)	3 (5.8)		0	3 (6.8)	
Positive radial margin, n (%)	0	0	–	0	0	–
pTNM stage, n (%)			0.293			0.343
I	32 (37.7)	14 (26.9)		18 (40.9)	13	
II	30 (35.3)	18 (34.6)		15 (34.1)	(29.6)	
III	22 (25.9)	20 (38.5)		11 (25)	14 (31.8)	
IV	1 (1.2)	0		10 (22.7)	17 (38.6)	
Adjuvant chemotherapy (stage II or III), n (%)	19 (36.5)	14 (36.8)	1	10	11 (25)	1
*Recurrence*						
Liver	5	4		1	4	
Lung	2	1		1	1	
Peritoneum	1	2		0	2	
Distant lymph node	2	0		2	0	
Other organ	1	1		1	1	

### Comparison of long-term oncological outcomes between groups

**Fig. 2:**
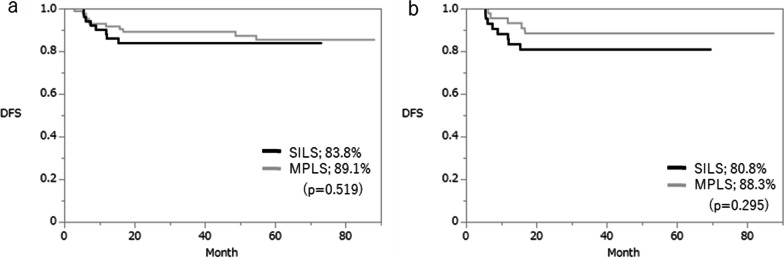
Kaplan–Meier analysis of disease-free survival rates between groups before (**a**) and after (**b**) matching

**Fig. 3:**
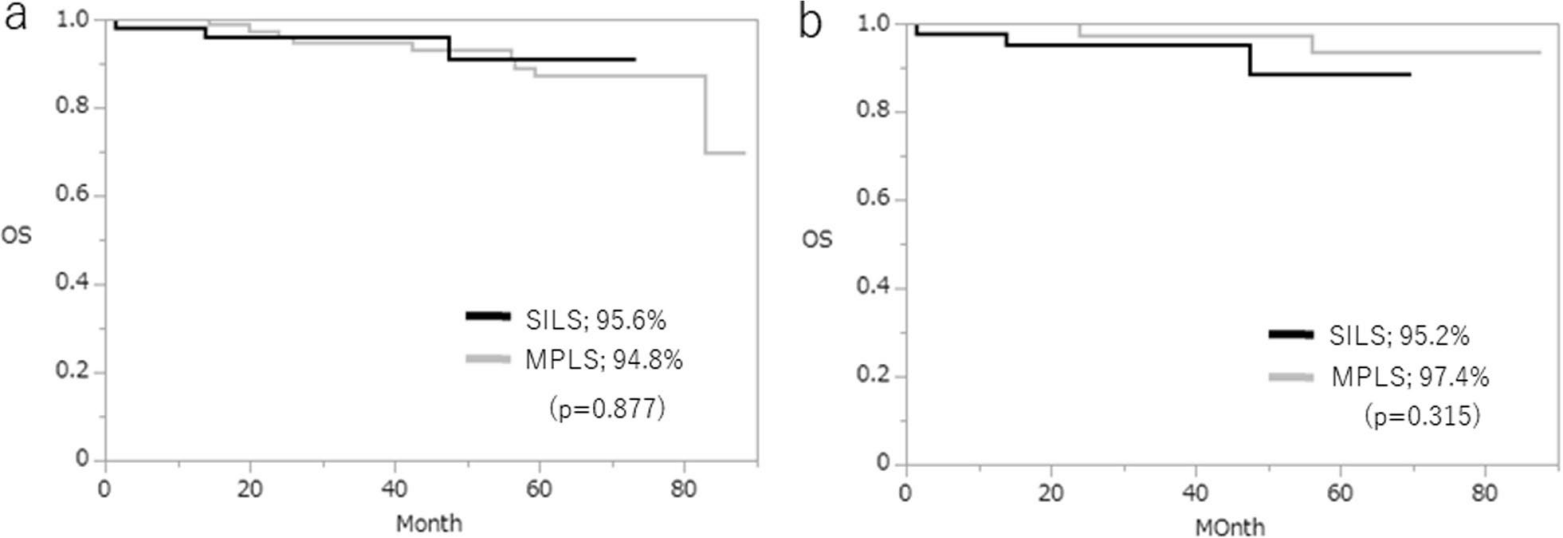
Kaplan–Meier analysis of overall survival rates between groups before (**a**) and after (**b**) matching

The median follow-up was 41 months (range, 22–53 months) in the SILS group and 60 months (range, 37 − 34 months) in the MPLS group (p = 0.001). In the overall cohort, 11 patients in the MPLS group experienced recurrence (liver, n = 5; lung, n = 2; peritoneum, n = 1; distant lymph node metastases, n = 2; adrenal glands, n = 1), compared to 8 patients in the SILS group (liver, n = 4; lung, n = 1; peritoneum, n = 2; ovary, n = 1). The 3-year disease-free survival rate was 89.1% in the MPLS group and 83.8% in the SILS group (Fig. 2a), and the 3-year overall survival rate was 94.8% in the MPLS group and 95.6% in the SILS group (Fig. 3a). After matching, the 3-year disease-free survival rate was 88.3% in the MPLS group and 80.8% in the SILS group (Fig. 2b), and the 3-year overall survival rate was 97.4% in the MPLS group and 95.2% in the SILS group (Fig. 3), showing no significant differences between groups.

Table 5 shows the results of uni- and multivariate analyses of clinical factors for disease-free and overall survival in the overall cohort. We identified pathological T4 stage (odds ratio [OR], 7.160; 95% CI (confidence interval) 2.713–18.894) and lymph node metastasis (OR 5.219; 95% CI, 1.654–16.465) as significant independent determinants of disease-free survival. Multivisceral resection (OR 7.424; 95% CI, 1.874–29.411) and pathological T4 stage (OR 6.682; 95% CI, 1.774–25.171) represented significant independent determinants of overall survival.

**Table 5:** Uni- and multivariate analyses of clinical factors predicting long-term oncological outcomes in the overall cohort

Variables	Disease-free survival		Overall survival	
	Univariate P value	Multivariate * P* value, HR (95% CI)	Univariate P value	Multivariate *P* value, HR (95% CI)
Age, > 75years	0.386		0.374	
Sex, male	0.069	0.341 1.579 (0.617–4.044)	0.524	
Approach, SILS	0.519		0.877	
Extent of lymph node dissection, D2	0.832		0.222	
Multivisceral resection, yes	0.322		< 0.001	0.004 7.424 (1.874–29.411)
Tumor invasion, pT4	< 0.001	< 0.001 7.160 (2.713–18.894)	< 0.001	0.005 6.682 (1.774–25.171)
LN metastasis, positive	< 0.001	0.005 5.219 (1.654–16.465)	0.167	0.956 1.036 (0.289–3.715)
Number of harvested LN <12, yes	0.948		0.228	
Postoperative complication, yes	0.786		0.455	

## Discussion

The present study appears to be the first to compare clinical outcomes between SILS and MPLS for DCC. The results suggest that, in selected patients, SILS for DCC can be performed safely and feasibly (as per the 98.1% SILS completion rate) and yields adequate short-term surgical outcomes (e.g., 25.0% morbidity, 0% mortality) in the entire patient cohort. In terms of oncological outcomes, we achieved a 100% R0 resection rate, and satisfactory 3-year disease-free and overall survival rates in patients with DCC who underwent SILS in both the entire patient cohort and matched cohort.

In this study, SILS was successfully performed in 98.1% of patients, including 17 patients (32.7%) with a history of prior abdominal surgery. In a previous systematic review of SILS for colorectal cancer [17], the rate of conversion to open surgery was 0.9, and 13.3% of patients who underwent SILS procedures required insertion of an additional port to allow completion of the operation. Those findings were comparable with the present results. Median operative time was about 10 min longer in the SILS group both before and after matching, but this was not significant. In previous studies [18–23], the operative time of laparoscopic surgery for splenic flexure colon cancer ranged from 178 to 283 min, comparable with our findings regardless of SILS or MPLS. Generally, SILS is technically limited due to factors such as instrument crowding, inline positioning of the laparoscope, and insufficient triangulation [2, 3], especially in mobilization of the splenic flexure and regional lymph node dissection; these issues would contribute to extend the operative time. The volume of blood loss was significantly lower in the SILS group than in the MPLS group for both the entire cohort (*p * < 0.001) and matched cohort (*p * = 0.011). In our study, patients with DCC who underwent MPLS were enrolled between April 2008 and December 2018, while SILS was performed for patients from March 2011 to December 2018. This difference in historical background may have affected the results. Other perioperative outcomes, including multivisceral resection rate and postoperative complications, did not differ between groups, and were comparable with findings from previous studies [5–10]. Although this study analyzed only 137 patients and used a retrospective design to investigate patients from two hospitals, our results with SILS showed high reliability in terms of successful completion rate and perioperative outcomes in patients with DCC.

In cancer treatment, oncological clearance must take precedence over cosmetic advantages or reduced invasiveness. The number of harvested lymph nodes was significantly larger in the SILS group than in the MPLS group for the entire patient cohort (*p * < 0.001) and matched cohort (*p * = 0.043). In this study, the D3 lymph node dissection rate was significantly higher for the SILS group than for the MPLS group (*p * = 0.021) in the entire patient cohort, and also tended to be high in the matched cohort (*p * = 0.085). This may have affected the difference in number of harvested lymph nodes. The oncological outcomes, including proximal margin, distal margin, and residual tumor status, were comparable to those from randomized control trials comparing open and MPLS for colorectal cancer [24–27], as well as those comparing MPLS and SILS for colon cancer [5–10]. In the present study, the 3-year disease-free survival rate, 3-year overall survival rate, and recurrence pattern did not differ between groups. Our results are comparable to findings from previous studies that have reported long-term outcomes of SILS for colon cancer [28, 29] or oncological outcomes of DCC [30, 31]. Multivariate analyses showed that the surgical approach performed was not associated with disease-free or overall survival, whereas pathological T4 and lymph node metastasis were significant independent determinants of disease-free survival. In our study, 9 patients underwent multivisceral resection, including five with tumor infiltration, three with tumor-associated abscess, and one with adhesions. Multivisceral resection was a significant independent determinant of overall survival, which may have been due to tumor-associated abscess.

Several limitations warrant consideration when interpreting the results of this investigation. First, data were obtained retrospectively, and the sample size was small. Second, this study showed bias in terms of the dates of operations. To overcome this limitation, we matched cases using several clinical variables, balancing groups and reducing selection bias. However, the potential for selection bias remains, despite the propensity score-matching. Third, BMI in our cohort was typical of a Japanese population, and may have significantly affected the surgical results of SILS. Fourth, the duration of follow-up was significantly shorter in the SILS group (41 months) than in the MPLS group (60 months, *p * = 0.001). Long-term oncological outcomes and rates of later complications such as umbilical incisional hernia thus could not be assessed in the SILS group. Despite these limitations, we consider that this analysis using propensity score-matching confirmed SILS as a safe and feasible option for DCC. Further analyses are required to validate our results, and to evaluate the long-term oncological outcomes in future randomized clinical trials.

## Conclusion

SILS is a safe, feasible method that can provide satisfactory oncological outcomes in selected patients with DCC.

## Data Availability

The datasets generated and/or analyzed during the current study are not publicly available, due to the privacy of the enrolled subjects, but may be provided by the corresponding author upon reasonable request.
